# Comparative analysis of 1^st^, 2^nd^, and 4^th ^year MD students' attitudes toward Complementary Alternative Medicine (CAM)

**DOI:** 10.1186/1756-0500-1-84

**Published:** 2008-09-17

**Authors:** Christopher P Riccard, Michele Skelton

**Affiliations:** 1 Department of Integrative Health Science, Stetson University, DeLand, Florida, USA

## Abstract

**Background:**

To identify and report the attitudes and beliefs of 1^st^, 2^nd^, and 4^th ^year medical students toward complementary alternative medicine (CAM).

**Methods:**

The previously validated and reliability tested CHBQ was administered to medical students attending the University of South Florida School of Medicine.

**Results:**

Significant changes were found between both 1^st ^(46.0 ± 7.7) and 4^th ^(37.8 ± 15.7) year students and 2^nd ^(48.3 ± 7.8) and 4^th ^(37.8 ± 15.7) year students. No significant difference was found between 1^st ^(46.0 ± 7.7) and 2^nd ^(48.3 ± 7.8) year students. When comparing scores based on gender, a significant difference was present between males (41.2 ± 12.2) and females (46.1 ± 11.0).

**Conclusion:**

CHBQ scores were significantly more positive in both 1^st ^and 2^nd ^year medical students in comparison with 4^th ^year student's scores. These findings suggest that as student exposure to allopathic techniques and procedures increases during the last year of medical school, their attitudes toward CAM decrease. Females were also significantly more likely to have stronger positive attitudes toward CAM than males, though both genders represented an overall positive attitude toward CAM.

## Background: CAM theories and training

Complementary alternative medicine (CAM), also referred to as "complementary therapies", describes those diagnostic and therapeutic techniques outside the realm of what is considered conventional medicine in the United States [1]. Because the definition of "complementary therapies" differs from country to country and changes throughout time, the exact boundary between commonly accepted therapies and those therapies considered complementary varies and is continually changing [2]. Surveys report, that among the general United States population, about 72 million Americans used at least one form of CAM in both 1997 and 2002, showing a steady need for knowledgeable CAM practicioners [3]. Included among those therapies classified as complementary are acupressure, acupuncture, applied kinesiology, aroma therapy, herbal medicine, homoeopathy, hypnosis, massage, meditation, naturopathy, nutritional therapy, reflexology, shiatsu, and yoga [2]. The development of unifying criteria for classifying therapies as complementary remains difficult due to the large number of varying therapies included in this classification [2]. However, because CAM use remains high (about 72 million American's in both 1997 and 2002) requiring allopathic physicians to become more knowledgeable about its therapies, research in the areas of allopathic physician's attitudes toward CAM, its availability, and its treatment practices is needed and a possible change in medical curriculums may need to occur. As a means to improve CAM availability and education in the United States, the National Center for Complementary and Alternative Medicine at the National Institutes of Health funded 15 educational projects focused on improving CAM knowledge at 14 different medical institutions [4]. Outcomes of the program, indicate a need to include CAM education into current conventional medical education programs so that health professionals can correctly inform patients of the benefits and risks associated with various CAM methodologies [4].

## CHBQ

The CAM Health Belief Questionnaire (CHBQ) is useful in measuring medical students' attitudes toward alternative medicinal theories and practices. Composed of 10 items, the CHBQ requires little time to complete and the survey's complexity is minimized [5]. The CHBQ has previously been both validity and reliability tested in its analysis and ability to score student's attitudes toward CAM [5,6]. Proper administration of the CHBQ requires that the survey be administered in full (10 questions) and an overall score be calculated; questions and scores cannot be examined individually to measure attitudes toward CAM. This survey uses a 7 point likert scale, 1 meaning absolutely disagree and 7 meaning absolutely agree [5]. Three items are negatively worded in order to prevent the respondent from consistently answering each question using only a portion of the rating scale provided [5]. A maximum score of 70 represents a positive attitude toward CAM modalities, while a score of 35 corresponds with a neutral attitude [6].

## Review of gender differences

While research regarding attitudes toward alternative medicinal theories is limited, some studies do show that gender does play a role in one's favorability of CAM [7,8]. After administering a brief survey similar to the CHBQ to 662 1^st^, 2^nd^, and 3^rd ^year medical students at the University of Birmingham's Medical School, results indicated that females showed a significantly more positive attitude toward CAM than did males [8]. A second study, in which a modified version of the IMAQ was used for data collection, was administered at medical schools in the United Kingdom, New Zealand, Canada, the United States, Hong Kong, and China. A significant gender difference was reported between males and females. Of the 639 students studied, female students had higher positive attitudes toward the theories of holism and the overall effectiveness of treatments involving CAM than did males [7].

## Review of student differences

Studies that examined class level differences vary in outcome and groups surveyed. According to a survey by Baugniet et. al. (2000) given to 61 4^th ^year medical students, 35.4% agreed or strongly agreed that "CAM is a useful supplement to regular medicine" [9]. This same study also found that medical students were more likely to place a higher emphasis on scientific-based data (randomized clinical trials and animal studies) as opposed to "anecdotal" beliefs when supporting the usefulness and outcomes of CAM therapies [9]. Overall, medical students received the least amount of training in CAM when compared with other therapeutic fields including physiotherapy, occupational therapy, nursing, and pharmacy [9].

An earlier study of third year medical students at the University of South Florida (USF) reported that approximately one third the population (28 of 100) surveyed associated the effects of CAM therapy with those of a placebo while 35 participants remained neutral and 37 disagreed [10]. Ninety-nine percent of respondents agreed that public CAM demand and interest was increasing [10]. Half the survey respondents (> 50%) reported believing in the usefulness of 7 CAM therapies including acupuncture (64%), chiropractic (56%), herbology (73%), hypnosis (62%), meditation (90%), massage (87%) and spiritual healing (61%) [10]. A majority (> 50%) of this same cohort reported that they would "refer" or "encourage" the use of two CAM therapies, meditation and massage [10].

At the Georgetown University School of Medicine, 266 1^st ^and 2^nd ^year students participated in a study examining CAM and its position in medical education and practice. Chaterji et. al. (2007) reports that 91% of students surveyed agreed that conventional medicine could benefit from CAM teachings and techniques [11]. Furthermore, 85% of those surveyed agreed that knowledge about CAM would play an important role in their futures as practicing physicians [11]. The results of this study indicate a similar belief in and toward CAM among 1^st ^and 2^nd ^year students, as on both statements previous, they were in agreement.

Furnham et. al. (2003) reports that of 311 1^st ^and 3^rd ^year medical students surveyed, 27.8% of respondents are knowledgeable in 18 CAM therapies, 40.5% would refer a patient/client for CAM, and 51.4% believe CAM to be an effective means of therapy [12]. Differences between the two class levels (172 1^st ^year and 139 3^rd ^year students) were analyzed indicating that 1^st ^year medical students believed CAM to be more effective than 3^rd ^year medical students [12]. Similarly, 1^st ^year students demonstrated a greater desire for training in CAM therapies than did 3^rd ^year students [12].

One study incorporating the CHBQ administered to a total of 355 medical students (170 1^st ^year and 185 2^nd ^year) averaged a mean score of 47.8, equal to a slight positive attitude toward CAM [6]. A small cohort was also surveyed during its 3^rd ^year. Year 1 medical students had a mean score of 46.4, 2^nd ^year students averaged 47.4, and 3^rd ^year students averaged 48.3, representing no significant change between 1^st ^and 3^rd ^year medical students [6].

The previous data (not limited to that reviewed in this article) regarding attitudinal differences between MD class levels (i.e. 1^st^, 2^nd^, 3^rd^, and 4^th ^year students) provides conflicting results. Thus, it is the purpose of this study to obtain another set of data to compare with previous studies. Based on these findings and the effectiveness of the methods and CHBQ survey, a longitudinal study that can be administered nationally will be designed and its results reported. The specific data provided by this study will also allow variations in attitudes toward CAM between medical institutions and geographical areas to be compared (for example: data from USF vs. data from a northern institution). Furthermore, in examining the changes in attitude toward CAM that occur throughout one's medical education, medical institutions can redesign current or develop new curricula to accommodate such changes.

## Methods and measures

### Respondents and their characteristics

The final collected sample included 95 respondents. Broken down by class, 36 were in their 1^st ^year of medical school, 29 in their 2^nd ^year, and 30 in their 4^th ^year (response rate for each year was 30%, 24.2%, and 25.2% in respective order when compared to original entering class size). The survey was administered to 1^st ^year students at the beginning of their 1^st ^year of medical education (October 2007), 2^nd ^year students during their first month of their respective year in school (October 2007) and 4^th ^year students during their final month of medical school (April 2007). Originally, the study sought out to examine MD students upon their entrance (1^st ^year) and exit (4^th ^year) of medical school, however, access was gained to 2^nd ^year students which accounts for their inclusion in this study. Because the 2^nd ^year students were in the beginning of their 2^nd ^year, and only had 1^st ^year educational backgrounds, they were included in this sample. Third year MD students were beginning their clinical rotations, thus, access to their student cohort was limited and they were excluded. All respondents were students at the USF School of Medicine. Broken down by gender, 40% (38) of the respondents were male; 60% (57) were female.

### CHBQ distribution and scoring

In conducting this cross-sectional study, the previously validated and reliability tested Cam Health Belief Questionnaire (CHBQ) was utilized. The 10 question survey which measures medical students' attitudes toward CAM was administered through a web-based survey program that first, second, and fourth year University of South Florida (USF) MD students were granted access to through a standardized group email sent out to the student population. Students were permitted ten days to complete the survey and participation was optional. All participants were required to provide consent prior to starting the survey; confidentiality was maintained. The survey was graded using a 7-point likert scale; 1 meaning absolutely disagree and 7 meaning absolutely agree. Three questions (#s 6, 7, and 8) are negatively worded and scaled in reverse. A maximum score of 70 correlates with a strongly positive attitude toward CAM, a score between 69-36 representative of a positive attitude, and a score of 34-11 correlates negatively with attitudes toward CAM and a 10 is the minimum score possible (representative of the least favorable attitude toward CAM; a score of exactly 35 is neutral. A one-way ANOVA (using SPSS software) was used to examine mean CHBQ scores between 1) 1^st ^year, 2^nd ^year, and 4^th ^year MD students and 2) between male and female students (P ≤ .05) [see Table 1].

**Table 1:** CHBQ items [5]

**1)**	The physical and mental health are maintained by an underlying energy or vital force.
**2)**	Health and disease are a reflection of balance between positive life-enhancing forces and negative destructive forces.
**3)**	The body is essentially self-healing and the task of a health care provider is to assist in the healing process.
**4)**	A patient's symptoms should be regarded as a manifestation of general imbalance or dysfunction affecting the whole body.
**5)**	A patient's expectations, health beliefs and values should be integrated into the patient care process.
**6)**	Complementary therapies are a threat to public health. (reverse scaled)
**7)**	Treatments not tested in a scientifically recognized manner should be discouraged. (reverse scaled)
**8)**	Effects of complementary therapies are usually the result of a placebo effect. (reverse scaled)
**9)**	Complementary therapies include ideas and methods from which conventional medicine could benefit.
**10)**	Most complementary therapies stimulate the body's natural therapeutic powers.

## Results

### CHBQ scores

The mean scores for 1^st^, 2^nd^, and 4^th ^year students were 46.0 ± 7.7, 48.3 ± 7.8, and 37.8 ± 15.7 [see Table 2]. Significant changes were found between both 1^st ^and 4^th ^year students (p = 0.012) and 2^nd ^and 4^th ^year students (p = 0.002). No significant difference was found between 1^st ^and 2^nd ^year students (p = 0.683). When comparing scores based on gender, females (46.1 ± 11.0) scored significantly higher (p = 0.046) than males (41.2 ± 12.2).

**Table 2:** Mean CHBQ scores and standard deviations

**Group**	**Mean**	**Standard Deviation**	**Number**
**1**^st^	46.0	± 7.7	36
**2**^nd^	48.3	± 7.8	29
**4**^th^	37.8	± 15.7	30
**Males**	41.2	± 12.2	38
**Females**	46.1	± 11.0	57

## Discussion

### Class level differences

Similar to previous studies involving medical students' attitudes toward CAM [6,12], this study indicates significantly more positive attitudes toward CAM in both 1^st ^(46.0 ± 7.7) and 2^nd ^(48.3 ± 7.8) year medical students as compared to 4^th ^year (37.8 ± 15.7) student's attitudes. One study by Lie et. al. (2006), using the CHBQ, found comparable results to those being reported [6]. A cohort of 170 1^st ^year and 185 2^nd ^year medical students shared CHBQ mean scores of 46.4 and 47.4 respectively. The mean CHBQ score for 3^rd ^year medical students, 48.3, indicated no significant change [6]. The scores reported by Lie et. al. (2006) from both 1^st ^and 2^nd ^year medical students are consistent with the scores being reported in this study. Another study by Furnham et. al. (2003) reports that 172 1^st ^year medical students believe CAM to be more effective than 139 3^rd ^year medical students [12], indicative of a change in attitude toward CAM between the 1^st ^and 3^rd ^years of medical school. This study supports these findings [12].

Although this study found all three class levels representative of a positive attitude toward CAM, the scores of 1^st ^and 2^nd ^year students' attitudes are significantly higher, and thus, more positive toward CAM while 4^th ^year students' attitudes represent a slightly positive attitude toward CAM (based on a score of 35 representing a neutral attitude toward CAM). One primary explanation for this change in attitude can be attributed to the methods in which instruction is given during the first two years of medical school compared to the second two years of medical school. The 1st and 2nd years of the USF curriculum include primarily classroom based lecture, consisting of subjects including but not limited to ethics, humanities, anatomy, physiology, behavioral medicine, pathology, pharmacology, and clinical problem solving. As students enter their fourth year, however, the role of classroom instruction decreases while the amount of time spent in specific medical rotations/clinical increases. It was the hypothesis of this study, that as classroom based learning decreases and student exposure to allopathic techniques and procedures increases (during the last year of medical school), medical students attitudes toward and beliefs in CAM will decrease. The changes in mean CHBQ scores support this hypothesis.

### Gender differences

Upon comparing mean scores based on gender, females (46.1 ± 11.0) had significantly more positive attitudes toward CAM than males (41.2 ± 12.2), though both genders represented an overall positive attitude toward CAM. These findings are consistent with results from other studies in which medical students' attitudes toward CAM based on gender were examined [7,8]. One study using a brief survey similar to the CHBQ at the University of Birmingham's Medical School, indicated that females showed a significantly more positive attitude toward CAM than did males [8]. A second study, using a modified version of the IMAQ reported a significant difference in attitudes toward CAM between males and females; female students had higher positive attitudes toward the theories of holism and the overall effectiveness of treatments involving CAM than did males [7]. These results indicate that females are more open to the ideas and theories supporting alternative forms of medicine than males.

### Limitations and need for further research

One limitation of this study may be that the attitudes of individual groups entering medical school each year are already more positive toward CAM than the attitudes of those students who entered medical school in previous years. This factor can be discredited as a limitation by readministering the CHBQ to students during each year of their medical training. Furthermore, the health industry in Florida is very different from the current health industries found in both Western and Northern states, a factor which may have also limited the results of this study. Included as part of Florida's health industry, are two specific CAM programs directed toward students. As a minimal limitation, the authors recognize both the AMSA Leadership Retreat for CAM (which has been held in Florida) and the HEART program (at the University of Florida College of Medicine) as minor limitations that may have influenced student's attitudes toward CAM, however, this inference is strictly theoretical. Furthermore, programs similar to these are not limited to Florida (specifically the southeast region) and similar programs are found throughout the country. The authors also recognize the low response rate of medical students in completing the web based CHBQ. However, in a comparative study of web and mail survey response rates, it has been reported that web response rates (with email notification only) are 10% lower than mail survey response rates (Kaplowitz, Hadlock and Levin. A Comparison of Web and Mail Survey Response Rates. Public Opinion Quarterly, Vol. 68 No. 1 Pp. 94–101, 2004). This study reports a 21% response rate for email notification only survey instruments. The response rates for our study (email notification only) are higher than the response rates reported in the Kaplowitz, et. al (2004) study. The most important limitation of this study remains that it was conducted at only one medical institution (The University of South Florida) and that its results may not be representative of the attitudes of medical students nationally.

Despite these limitations, the significant difference in CHBQ scores reported in this study between both 1^st ^and 2^nd ^year medical students when compared with 4^th ^year medical students gives strong cause for confidence in an increasing interest and positive attitude (as all mean scores represented a positive attitude) toward CAM. This level of interest and the extent to which it changes during medical training, however, remains to be studied, as a longitudinal nationwide survey involving other medical institutions would be more suited for this purpose.

## Competing interests

The authors declare that they have no competing interests.

## Authors' contributions

MS carried out the survey's administration and statistical analysis, CR conceived the study, participated in its design, and drafted the manuscript. All authors read and approved the final manuscript.
